# Assessing the Cardiovascular Effects of Levothyroxine Use in an Ageing United Kingdom Population (ACEL-UK): Cohort Study

**DOI:** 10.1210/clinem/dgaf208

**Published:** 2025-04-03

**Authors:** Mia Holley, Salman Razvi, Ian Maxwell, Rosie Dew, Scott Wilkes

**Affiliations:** School of Medicine, Faculty of Health Sciences and Wellbeing, University of Sunderland, Sunderland SR1 3SD, UK; Translational and Clinical Research Institute, Newcastle University, Newcastle-upon-Tyne NE1 3BZ, UK; School of Medicine, Faculty of Health Sciences and Wellbeing, University of Sunderland, Sunderland SR1 3SD, UK; School of Medicine, Faculty of Health Sciences and Wellbeing, University of Sunderland, Sunderland SR1 3SD, UK; School of Medicine, Faculty of Health Sciences and Wellbeing, University of Sunderland, Sunderland SR1 3SD, UK

**Keywords:** aged, cardiovascular, general practice, hypothyroidism, levothyroxine, osteoporosis, thyrotropin, thyroxine

## Abstract

**Context:**

Thyrotropin (TSH) levels tend to rise with age, but standard reference intervals do not reflect this, potentially leading to overdiagnosis of subclinical hypothyroidism (SCH) and excessive levothyroxine (LT4) prescriptions in older adults.

**Objective:**

This work aimed to compare outcomes in adults older than 50 years with SCH who were either prescribed or not prescribed LT4.

**Methods:**

A retrospective cohort study was conducted using data from UK Primary Care patients from the Health Improvement Network. The primary outcome was cardiovascular (CV) events (angina, myocardial infarction, peripheral vascular disease, stent procedures, or stroke). Secondary outcomes included bone events (fragility fractures or osteoporosis) and all-cause mortality. Time-varying hazard ratios (HRs) adjusted for relevant factors were estimated.

**Results:**

This study included 53 899 patients (baseline median age 67 years (interquartile range [IQR]: 59-76 years); 68.5% female; median TSH 4.6 mU/L (IQR: 4.1-5.4 mU/L). Median follow-up duration was 10 years (IQR: 5.5-10.0 years). Of these, 19 952 (37%) received LT4 and 33 947 (63%) did not. LT4 therapy showed a protective effect against CV events (HR: 0.91; 95% CI, 0.87-0.97; *P* < .001) but increased risk of bone events (HR: 1.21; 95% CI, 1.14-1.28; *P* < .001) and all-cause mortality (HR: 1.17; 95% CI, 1.13-1.22; *P* < .001).

**Conclusion:**

Our data suggest that LT4 therapy in older individuals with SCH is associated with a trade-off between the potentially beneficial effect on CV risk and the deleterious relationship with bone health and mortality risk. These risks need to be considered, mitigated, and discussed when LT4 therapy is being deliberated in older patients with SCH.

Hypothyroidism is a widespread chronic condition arising from insufficient production of thyroid hormones (1). In the United Kingdom, hypothyroidism affects approximately 5% to 10% of the general population (2), with a higher prevalence among females and individuals older than 60 years (3-5). Subclinical hypothyroidism (SCH) is defined by elevated serum thyrotropin (TSH) levels and normal levels of free thyroxine (fT4) (1, 6).

Mildly elevated TSH levels become more prevalent with age. The National Health and Nutrition Examination Survey in the United States studied 16 533 adults without thyroid disease, revealing a significant proportion of older adults with high TSH levels (>4.5 mU/L) (4). Similarly, the Thyroid Epidemiology, Audit, and Research Study (TEARS) in Scotland, with 153 127 participants, found that 97.5th percentile TSH levels steadily rose with age, reaching up to 5.9 mU/L in those older than 90 years (7). Longitudinal research further demonstrates a natural rise in TSH concentration with age, often reaching 97.5th percentile levels as high as 8.0 mU/L in those older than 90 years (8). Moreover, longitudinal studies indicate that TSH levels tend to rise with age, without significant changes in fT4 levels (8-10). Studies also suggest potential benefits associated with mildly elevated TSH levels (4.5-7.0 mU/L), such as improved mobility and lower mortality rates in older adults (11, 12). However, SCH is associated with higher cardiovascular (CV) risk; the risk increases significantly when TSH levels exceed 10.0 mU/L (12-15). These findings suggest adopting age-specific TSH intervals, in contrast to the 0.4 to 4.0/4.5 mU/L reference interval commonly used regardless of age.

It is widely acknowledged that patients diagnosed with overt hypothyroidism should receive levothyroxine (LT4) (16). In contrast, the management of SCH is uncertain due to insufficient reliable evidence (17). Overtreatment with thyroid hormones can lead to adverse health effects, such as increased CV risks and fractures (18, 19). Despite these risks, overtreatment remains common among older individuals, resulting in suppressed TSH levels when patients are prescribed LT4 (20). The European SCH guidelines specify that most adults should receive LT4 if their TSH levels exceed 10 mU/L and symptoms are present (21). However, adherence to these recommendations is not consistently followed (20).

Current research on the CV outcomes of LT4 treatment for SCH in older adults presents insignificant findings. A cohort study involving 1642 patients older than 70 years showed no difference in CV events between those treated with LT4 and untreated individuals (22). The Thyroid Hormone Therapy for Older Adults with Subclinical Hypothyroidism (TRUST) study, a randomized controlled trial including 737 adults aged 65 and older, found no statistically significant association between LT4 use and CV outcomes (hazard ratio [HR] 0.89; 95% CI, 0.47-1.69), although the trial was not adequately powered to detect this outcome (23). Pooled results from the TRUST study and another randomized controlled trial reflected these findings, indicating no considerable CV risk difference with LT4 treatment (24). A systematic review highlighted the lack of evidence on long-term CV and bone health outcomes in older adults with SCH older than 50 years, emphasizing the need for further research (25, 26). The Assessing the Cardiovascular Effects of Levothyroxine Use in an Ageing United Kingdom Population (ACEL-UK) study was designed to gather evidence to improve our understanding of this critical issue concerning the benefits and harms of LT4 therapy in older patients with SCH.

## Materials and Methods

### Study Design and Setting

A retrospective cohort study was conducted using observational data from The Health Improvement Network (THIN). THIN contains electronic health care records of approximately 6% of the UK population, derived from 850 UK general practices. Its data collection began in 2003, with information dating back to 1994. The data set holds anonymized longitudinal medical records of 19.4 million patients, with 2.8 million active patients (27). The protocol for this study was published in November 2023 (28).

### Study Population

Data were extracted for patients who were older than 50 years on January 1, 2006, with at least 1 TSH reading exceeding 4.0 mU/L between January 1, 2006, and January 1, 2022. The inclusion and exclusion criteria were then applied to this data set.

#### Inclusion criteria

1. Patients with a baseline TSH level between 4.0 mU/L and 10.0 mU/L (if prescribed LT4), or a median TSH level within this range during the follow-up period (if not prescribed LT4).
2. Patients with a baseline fT4 level between 12.0 pmol/L and 22.0 pmol/L (if prescribed LT4), or a median fT4 level within this range during the follow-up period (if not prescribed LT4).
3. Patients registered in the THIN database between January 1, 2006, and January 1, 2016.

#### Exclusion criteria

1. Patients with a baseline (if prescribed LT4) or median (if not prescribed LT4) TSH level below 4.0 mU/L or above 10.0 mU/L.
2. Patients with a baseline (if prescribed LT4) or median (if not prescribed LT4) fT4 level below 12.0 pmol/L or above 22.0 pmol/L.
3. Patients with history of thyroid cancer, pituitary disease, or hyperthyroidism.
4. Patients who had received thyroid surgery or radioiodine treatment.
5. Patients prescribed liothyronine, amiodarone, or lithium.
6. Patients with a baseline diagnosis of angina, myocardial infarction, peripheral vascular disease, coronary artery stent, or stroke (for CV outcomes only) or a baseline diagnosis of fragility fracture or osteoporosis (for bone health outcomes only).

Search terms were focused on International Classification of Diseases, Tenth Revision (ICD-10) codes: E05 for hyperthyroidism, C73 for thyroid cancer, E22 to E24 for pituitary disease, I20 for angina, I21 to I23 for myocardial infarction, I60 to I64 for stroke, I70 to I79 for peripheral vascular disease, M80 to M81 for osteoporosis, and M84.4, S32, S52.5, or S62 for fragility fractures. Codes relating to Raynaud disease, vibration syndrome, hereditary diseases, naevus, telangiectasia, post radiological, or Williams-Campbell syndrome were excluded from peripheral vascular disease codes. Also, fractures on digits or pathological fractures were not included in the search for fragility fractures. Notably, ICD-10 codes do not encompass treatments or surgery. Therefore, treatment terms were based on treatment names, and surgery terms were based on read codes. As a result, read codes were used to categorize patients who underwent a stent procedure based on the code list res12: percutaneous transluminal coronary angioplasty (29). Ethnicity was categorized according to the Census 2021 ethnicity classifications. THIN provided information on whether a patient's sex was assigned male or female at birth. THIN records a patient's smoking status as never smoked, used to smoke, or currently smokes. In cases where multiple smoking codes were presented for a patient, the most recent code was used for analysis.

### Treatment Strategy

The study comprised two groups: those prescribed LT4 and those not. Follow-up began from January 1, 2006, or from the first LT4 prescription, and ended at the earliest of death, outcome event, or January 1, 2016. Patients in the treatment group had exclusion criteria applied at the point of LT4 initiation.

### Outcome Measures

The primary outcome of this study was CV outcomes (angina, myocardial infarction, peripheral vascular disease, stent procedure, and stroke). The secondary outcomes of this study were bone health outcomes (osteoporosis and fragility fractures) and all-cause mortality. ICD-10 and read codes were used to identify outcomes. All-cause mortality was based on the recorded date of death. The first outcome was the outcome of interest.

### Statistical Analysis

Baseline characteristics were compared between groups. Frequency and percentages are presented for outcomes. The HRs are presented with their corresponding 95% CI and *P* values. A time-varying Cox proportional-hazards model was implemented to assess the association between LT4 and all 3 outcomes, adjusting both time-fixed and time-varying covariates, while ensuring that the proportional-hazards assumption was met. Age and sex were included as fixed covariates, while body mass index, Charlson comorbidity index (30), total cholesterol, hypertension, and smoking status were incorporated as time-varying covariates. Other comorbidities were not selected for adjustment, due to large levels of multicollinearity found with the Charlson comorbidity index. The time-varying covariates were updated at each follow-up interval. Each individual's follow-up time was divided into intervals during which these covariates were updated in the database, and these values were used to create intervals within the Cox model to capture changes in the covariates over time. Kaplan-Meier curves were displayed to visualize the survival probabilities. In this study, a statistical significance level of .01 was implemented to minimize type I error. This statistical significance level was chosen due to the 3 outcomes, using the Bonferroni correction at an initial statistical significance level of .05 (31). Ethnicity had more than 50% missing data, and therefore was not included in the analysis. Multiple imputation methods were used to address other missing data (supplemental data, Supplementary Table S1 (32)).

As serum TSH levels are known to rise with age, an age-specific TSH limit was used for one of the analysis. The 97.5th percentile TSH levels from the TEARS study were used for the various age groups and are presented in Table 1. Three primary groups were analyzed based on TSH levels (mU/L): group 1 (4.0-10.0), group 1a (4.0- TEARS 97.5th percentile), and group 1b (TEARS 97.5th percentile-10.0). Subgroup analyses were conducted by age group, sex, smoking status, and baseline fT4 levels (> or < the population median). Sensitivity analyses used TSH thresholds of 4.5 mU/L and 5 mU/L to account for variations across studies.

**Table 1.** List of 97.5th percentile thyrotropin levels by age from The Thyroid Epidemiology Audit and Research Study (7).

Age, y	97.5th percentile thyrotropin, mU/L
51-60	4.4
61-70	4.6
71-80	5.0
81-90	5.5
91+	5.9

## Results

There were 282 036 initial patient records received from THIN. After applying the a priori study criteria, 228 137 patients were removed (see supplemental data, Supplementary Fig. S1 (32)). There were 53 899 patients included in group 1 of the study for CV outcomes. Of those, 19 952 (37.0%) were prescribed LT4 and 33 947 (63.0%) were not prescribed LT4. There were 18 469 patients in group 1a, of whom 3486 (18.9%) were prescribed LT4 vs 14 983 (81.1%) not prescribed LT4. There were 35 430 patients in group 1b. Of those, 16 466 (46.5%) were prescribed LT4 vs 18 964 (53.5%) not prescribed LT4. The median (interquartile range [IQR]) follow-up time for CV outcomes was 10.0 (5.5-10.0) years for all 3 groups. There were more female than male patients, and most patients were aged 61 to 70 years, with few older than 91 years. Ethnicity was predominantly White, with a small proportion of Black patients. LT4 prescriptions were consistent across all smoking statuses. The treatment group had higher rates of comorbidities across all groups. Ethnicity was not considered for adjustment, with more than 40% missing (see supplemental data, Supplementary Table S1 (32)). Participant characteristics are shown in Table 2.

**Table 2.** Baseline characteristics of participants of the cardiovascular outcome study

Characteristic, N (%)	Group 1, treatment (n = 19 952)	Group 1, control (n = 33 947)	Group 1a, treatment (n = 3486)	Group 1a, control (n = 14 983)	Group 1b, treatment (n = 16 466)	Group 1b, control (n = 18 964)
**Sex**						
Female	15 588 (78.1)	21 350 (62.9)	2823 (81.0)	9502 (63.4)	12 765 (77.5)	11 848 (62.5)
Male	4364 (21.9)	12 597 (37.1)	663 (19.0)	5481 (36.6)	3701 (22.5)	7116 (37.5)
**Age, y**						
51-60	5141 (25.8)	10 991 (32.4)	495 (14.2)	3141 (21.0)	4646 (28.2)	7850 (41.4)
61-70	6687 (33.5)	10 309 (30.4)	895 (25.7)	4200 (28.0)	5792 (35.2)	6109 (32.2)
71-80	5185 (26.0)	8250 (24.3)	1125 (32.3)	4654 (31.1)	4060 (24.7)	3596 (19.0)
81-90	2533 (12.7)	3895 (11.5)	813 (23.3)	2631 (17.6)	1720 (10.4)	1264 (6.7)
91+	406 (2.0)	502 (1.5)	158 (4.5)	357 (2.4)	248 (1.5)	145 (0.8)
Median (lower quartile-upper quartile)	67 (60-76)	66 (59-75)	73 (65-81)	71 (62-79)	66 (60-75)	63 (57-71)
**Ethnicity**						
Asian	311 (1.6)	498 (1.5)	28 (0.8)	175 (1.2)	283 (1.7)	323 (1.7)
Black	52 (0.3)	127 (0.4)	8 (0.2)	49 (0.3)	44 (0.3)	78 (0.4)
Mixed	2937 (14.7)	5042 (14.9)	425 (12.2)	2138 (14.3)	2512 (15.3)	2904 (15.3)
Other	89 (0.4)	178 (0.5)	20 (0.6)	81 (0.5)	69 (0.4)	97 (0.5)
White	6197 (31.1)	10 840 (31.9)	1059 (30.4)	4674 (31.2)	5138 (31.2)	6166 (32.5)
No information	10 366 (52.0)	17 262 (50.8)	1946 (55.8)	7866 (52.5)	8420 (51.1)	9396 (49.5)
**Location**						
London	1219 (6.1)	2154 (6.3)	182 (5.2)	830 (5.5)	1037 (6.3)	1324 (7.0)
Midlands and East	2972 (14.9)	4285 (12.6)	482 (13.8)	1653 (11.0)	2490 (15.1)	2632 (13.9)
North	2889 (14.5)	4573 (13.5)	463 (13.3)	2068 (13.8)	2426 (14.7)	2505 (13.2)
Northern Ireland	1380 (6.9)	1963 (5.8)	242 (6.9)	984 (6.6)	1138 (6.9)	979 (5.2)
Scotland	2069 (10.4)	4523 (13.3)	385 (11.0)	2139 (14.3)	1684 (10.2)	2384 (12.6)
South	4626 (23.2)	8900 (26.2)	816 (23.4)	3700 (24.7)	3810 (23.1)	5200 (27.4)
Wales	3212 (16.1)	5226 (15.4)	647 (18.6)	2630 (17.6)	2565 (15.6)	2596 (13.7)
No information	1585 (7.9)	2323 (6.8)	269 (7.7)	979 (6.5)	1316 (8.0)	1344 (7.1)
**Smoker status**						
Smoker	1510 (7.6)	2548 (7.5)	206 (5.9)	985 (6.6)	1304 (7.9)	1563 (8.2)
Past smoker	6236 (31.3)	10 680 (31.5)	1063 (30.5)	4802 (32.0)	5173 (31.4)	5878 (31.0)
Non-smoker	12 174 (61.0)	20 525 (60.5)	2206 (63.3)	9101 (60.7)	9968 (60.5)	11 424 (60.2)
No information	32 (0.2)	194 (0.6)	11 (0.3)	95 (0.6)	21 (0.1)	99 (0.5)
**Comorbidities**						
Asthma	1936 (9.7)	1938 (5.7)	311 (8.9)	879 (5.9)	1625 (9.9)	1059 (5.6)
Chronic kidney disease	1895 (9.5)	73 (0.2)	266 (7.6)	39 (0.3)	1629 (9.9)	34 (0.2)
Chronic obstructive pulmonary disease	752 (3.8)	689 (2.0)	116 (3.3)	347 (2.3)	636 (3.9)	342 (1.8)
Dementia	108 (0.5)	36 (0.1)	31 (0.9)	19 (0.1)	77 (0.5)	17 (0.1)
Depression	2827 (14.2)	2238 (6.6)	409 (11.7)	963 (6.4)	2418 (14.7)	1275 (6.7)
Diabetes	1940 (9.7)	1305 (3.8)	343 (9.8)	695 (4.6)	1597 (9.7)	610 (3.2)
Dyslipidemia	2287 (11.5)	1782 (5.2)	410 (11.8)	905 (6.0)	1877 (11.4)	877 (4.6)
Heart disease	10 029 (50.3)	10 261 (30.2)	1783 (51.1)	5009 (33.4)	8246 (50.1)	5252 (27.7)
Hypertension	10 481 (52.5)	11 463 (33.8)	1986 (57.0)	5845 (39.0)	8495 (51.6)	5618 (29.6)
Rheumatoid arthritis	314 (1.6)	270 (0.8)	61 (1.7)	115 (0.8)	253 (1.5)	155 (0.8)
**Hormone levels**						
Low-normal fT4 levels	9761 (48.9)	17 122 (50.4)	1245 (35.7)	7014 (46.8)	8516 (51.7)	10 108 (53.3)
High-normal fT4 levels	10 191 (51.1)	16 825 (49.6)	2241 (64.3)	7969 (53.2)	7950 (48.3)	8856 (46.7)
TSH, median (lower quartile-upper quartile)	3.9 (2.3-5.0)	4.8 (4.3-5.5)	3.6 (2.7-4.4)	4.3 (4.1-4.5)	4.0 (3.0-5.1)	5.4 (4.9-6.2)

Group 1: Patients older than 50 years with a TSH level between 4.0 mU/L and 10.0 mU/L and a normal fT4 level.

Group 1a: Patients older than 50 years with a TSH level between 4.0 mU/L and the age-specific upper limit and a normal fT4 level.

Group 1b: Patients older than 50 years with a TSH level between the age-specific upper limit and 10.0 mU/L and a normal fT4 level.

Low-normal and high-normal fT4 levels are defined as above or below the median fT4 level.

Abbreviations: fT4, free thyroxine; TSH, thyrotropin.

For bone health outcomes, 225 158 patients were removed in line with the criterion. Of the 56 878 patients included in group 1, 21 347 (37.5%) were prescribed LT4 and 35 531 (62.5%) were not prescribed LT4. There were 19 686 patients included in the analysis of group 1a, of whom 3789 (19.2%) were prescribed LT4 and 15 897 (80.8%) were not prescribed LT4. Of the 37 192 patients in group 1b, 17 558 (47.2%) were prescribed LT4 and 19 634 (52.8%) were not prescribed LT4. There was a median (IQR) follow-up time of 10.0 (5.7-10.0) years for all 3 groups for bone health outcomes. A total of 221 249 patients were eliminated in line with the exclusion criteria for the cohort study looking at all-cause mortality outcomes. Of the 60 787 patients included in group 1, 23 435 (38.6%) were prescribed LT4 and 37 352 (61.4%) were not prescribed LT4. There were 21 098 patients in group 1a, of whom 4245 (20.2%) were prescribed LT4 and 16 853 (79.9%) were not prescribed LT4. There were 39 689 patients in group 1b; of these patients, 19 190 (48.4%) were prescribed LT4 and 20 499 (51.6%) were not prescribed LT4. There was a median (IQR) follow-up time of 10.0 (6.4-10.0) years for all 3 groups for all-cause mortality outcomes.

### Cardiovascular Outcomes

Incident CV events affected 12.3% of group 1 patients, 15.6% of group 1a, and 10.6% of group 1b (Table 3). Across all groups, the treatment group had lower CV event rates than the control group (9.2% vs 14.2% in group 1, 12.1% vs 16.5% in group 1a, and 8.6% vs 12.4% in group 1b). Group 1 exhibited significantly reduced HRs (HR 0.91; 95% CI, 0.87-0.97) (see Table 3). Group 1a presented similar results (HR 0.90; 95% CI, 0.81-0.90); group 1b did not reach statistical significance (HR 0.94; 95% CI, 0.88-1.00).

**Table 3.** Outcomes of the 10-year follow-up cohort study represented by raw numbers and adjusted time-varying hazard ratios

Outcome	Group 1			Group 1a			Group 1b		
	N (%), Treatment	N (%), Control	Time-varying hazard ratio	N (%), Treatment	N (%), Control	Time-varying hazard ratio	N (%), Treatment	N (%), Control	Time-varying hazard ratio
Cardiovascular	1836 (9.2%)	4809 (14.2%)	**0.91 (0.87, 0.97); *P* = .001**	421 (12.1%)	2466 (16.5%)	0.90 (0.81, 0.99); *P* = .039	1415 (8.6%)	2343 (12.4%)	.94 (0.88, 1.00); *P* = .067
Bone health	1686 (7.9%)	3330 (9.4%)	**1.21 (1.14-1.28); *P* < .001**	414 (10.9%)	1717 (10.8%)	**1.28 (1.15-1.42); *P* < .001**	1272 (7.2%)	1613 (8.2%)	**1.21 (1.12-1.30); *P* < .001**
All-Cause mortality	3774 (16.1%)	7502 (20.1%)	**1.17 (1.13-1.22); *P* < .001**	1053 (24.8%)	4090 (24.3%)	**1.20 (1.12-1.28); *P* < .001**	2721 (14.2%)	3412 (16.6%)	1.05 (1.00-1.10); *P* = .072

Group 1: Patients older than 50 years with a TSH level between 4.0 mU/L and 10.0 mU/L and a normal fT4 level.

Group 1a: Patients older than 50 years with a TSH level between 4.0 mU/L and the age-specific upper limit and a normal fT4 level.

Group 1b: Patients older than 50 years with a TSH level between the age-specific upper limit and 10.0 mU/L and a normal fT4 level.

Statistically significant associations are highlighted in bold.

Abbreviations: fT4, free thyroxine; TSH, thyrotropin.

### Bone Health Outcomes

In group 1, 8.8% of patients experienced bone health events, compared to 10.8% in group 1a and 7.8% in group 1b. In group 1 and group 1b, more events occurred in the control group than in the treatment group; group 1a had similar rates between groups. Group 1 revealed an HR of 1.21 (95% CI, 1.14-1.28; *P* < .001), indicating a statistically significantly increased bone outcome risk when prescribed LT4. Similarly, group 1a displayed an even higher HR of 1.28 (95% CI, 1.15-1.42; *P* < .001), and group 1b presented a statistically significant HR of 1.21 (95% CI, 1.12-1.30; *P* < .001) for bone health events.

### All-Cause Mortality

In total, 18.6% of patients in group 1 died, 24.4% in group 1a, and 15.5% in group 1b. Group 1 and group 1b had higher mortality in the control group compared to the treatment group (16.1% vs 20.1% in group 1, and 14.2% vs 16.6% in group 1b). In group 1a, mortality rates were similar between control and treatment groups (24.8% vs 24.3%). Outcome rates varied by age, sex, smoking status, and fT4 levels (see supplemental data and Supplementary Table S2 (32)). Group 1 had an HR of 1.17 (95% CI, 1.13-1.22; *P* < .001), indicating a significantly increased mortality risk with the use of LT4. Group 1a had a higher HR of 1.20 (95% CI, 1.12-1.28; *P* < .001), while group 1b had a nonsignificant HR of 1.05 (95% CI, 1.00-1.10; *P* = .072) (see Table 3). Unadjusted data are presented in the supplemental data and Supplementary Table S3 (32).

Adjusted time-varying HRs were also calculated for various subgroups (see supplementary data and Supplementary Table S4 (32)), which generally showed no association with bone health and CV outcomes but indicated an increase in mortality outcomes associated with treatment. Sensitivity analysis found similar outcomes but failed to reach statistical significance due to the reduced sample size (see supplemental data and Supplementary Table S5 (32)).

**Figure 1.**
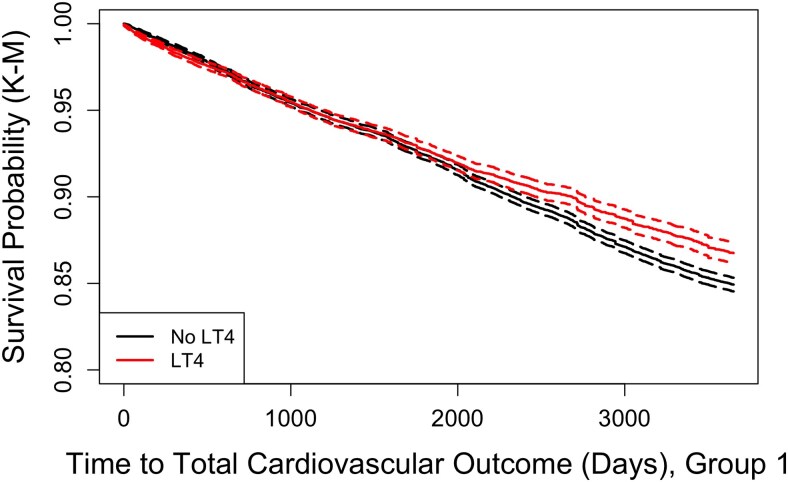
Kaplan-Meier plots illustrating survival probabilities over time for cardiovascular outcomes. Group 1: Patients older than 50 years with a thyrotropin level between 4.0 mU/L and 10.0 mU/L and a normal free thyroxine level.

The Kaplan-Meier plot for group 1 showed that the survival probability for CV outcomes was similar for both treatment groups up until approximately 1800 days (Fig. 1). However, after this, the treatment group had a higher survival rate. On the other hand, the Kaplan-Meier plot for group 1a and group 1b showed that the CV survival probability 95% CIs continually overlapped between both treatment groups. Furthermore, in group 1b, the Kaplan-Meier plot showed no difference in the survival curve, regardless of LT4 status. The Kaplan-Meier plots representing bone health and all-cause mortality outcomes showed that the survival probability was consistently better for the control group across all groups (see supplemental data and Supplementary Fig. S2 (32)).

## Discussion

This cohort study showed that LT4 treatment in older people with SCH is associated with improved CV health but higher risk of osteoporosis or fragility fractures and all-cause mortality. Additionally, those with TSH levels between 4.0 mU/L and the age-specific upper limit also demonstrated reduced CV risk, and an increased risk of bone health outcomes and all-cause mortality. The findings suggest that those treated with LT4 despite having age-specific TSH levels are at the greatest bone health and all-cause mortality risk. However, when split by subgroup an association is not prominent, likely due to the decreased sample size.

This cohort study greatly adds to the existing literature, being one of the most extensive study to date. A substantial strength of the study was the large sample size, which provided robust statistical power. Consistent and important findings concerning all outcomes were observed, supporting the reliability of the study. Moreover, the THIN database is representative of the UK population and has been proven to provide reliable clinical data from the vast number of studies published using the database, including one thyroid-related study (33, 34). Electronic health care record–based cohort studies provide numerous advantages but also present limitations. Limitations of this cohort study include data quality, biases, and generalizability to the population. Data quality issues, including missing information, were common among the data. For example, approximately 50% of patient records did not include ethnic information. Moreover, it is not known whether a patient collected their LT4 prescription, only if they were prescribed it. Additionally, some patients had fewer TSH levels recorded in the database than expected; this meant TSH could not be adequately adjusted for. These data quality issues highlight the inaccuracies in employing electronic health care records for research rather than clinical purposes. In addition, biases within the cohort study were present; misclassification bias resulted from inaccuracies in the data, and immortal time bias and selection bias resulted from the study design. Further, biochemical control was not assessed, leaving uncertainty about whether patients achieved optimal TSH levels. Unmeasured or unknown confounders may have influenced the outcomes, such as lifestyle factors or comorbidities not captured in the database. Additionally, while we used a uniform TSH reference range, variation in TSH reference intervals across general practices may have influenced the outcome. Another limitation is the lack of analysis on potential mechanisms underlying the observed CV benefit and the higher bone and mortality risks associated with treatment. For instance, it remains unclear whether the increase in bone events directly contributed to the higher mortality.

The observed reduction in CV risk associated with LT4 for older patients with SCH emphasizes the potential benefits of initiating LT4 treatment in this population. However, the increased risks of adverse bone health and all-cause mortality outcomes call for careful consideration when prescribing LT4 to older patients with slightly elevated TSH levels and normal fT4 levels. Given the findings of our study, clinicians should adopt a personalized approach for each patient dependent on demographics and comorbidities, prioritizing shared decision-making with the patient. As a result, the current clinical practice guidelines for LT4 prescription remain unchanged (21). The findings of our study suggest a potential association between bone health outcomes and all-cause mortality outcomes in an aging SCH population prescribed LT4. Future research into the prescribing of bone protection alongside LT4 should be considered.

## Data Availability

The data that support the findings of this study are available from THIN, a wholly owned subsidiary of Cegedim SA, which owns the proprietary rights to THIN data. Restrictions apply to the availability of these data, which were used under license for the present study and are not publicly available. Data are, however, available from the authors on reasonable request and with the permission of THIN.

## Abbreviations

ACEL-UK: Assessing the Cardiovascular Effects of Levothyroxine Use in an Ageing United Kingdom Population
CV: cardiovascular
fT4: free thyroxine
HR: hazard ratio
ICD-10: International Classification of Diseases, Tenth Revision
IQR: interquartile range
LT4: levothyroxine
SCH: subclinical hypothyroidism
TEARS: Thyroid Epidemiology, Audit, and Research Study THIN, The Health Improvement Network
TRUST: Thyroid Hormone Therapy for Older Adults with Subclinical Hypothyroidism
TSH: thyrotropin
